# Supplementary material for: Transcriptome analysis reveals role of transcription factor WRKY70 in early N-hydroxy-pipecolic acid signaling
Source: Plant Physiol. 2024 Oct 15;197(1):kiae544. doi: 10.1093/plphys/kiae544 (PMC11663584; doi:10.1093/plphys/kiae544)
Supplement: kiae544_Supplementary_Data [file kiae544_supplementary_data.zip › suppfigures.pdf]

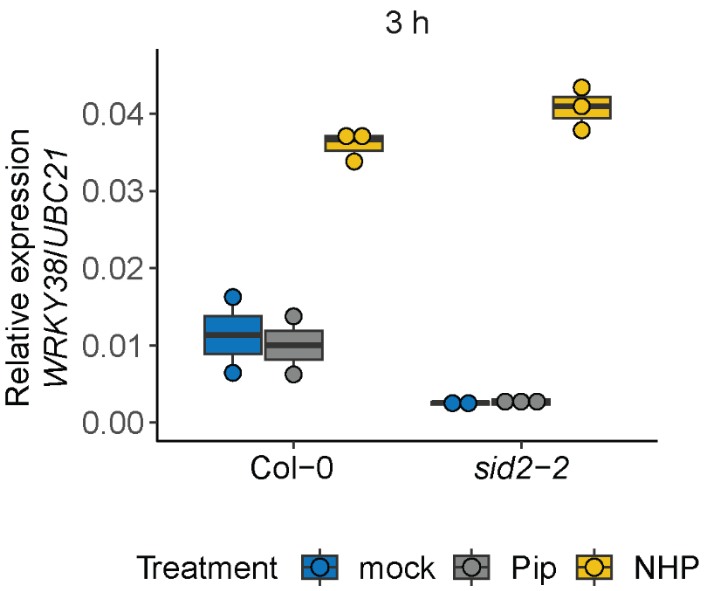

Supplementary Figure S1 NHP induced *WRKY38* expression is SA-independent. Hydroponically grown wild type (Col-0) and *sid2-2* (salicylic acid (SA) biosynthetic deficient mutant) Arabidopsis seedlings were treated at 10-days-post-germination (dpg) with MS medium (mock), 1 mM pipecolic acid (Pip), or 1 mM *N*-hydroxy-pipecolic acid (NHP) then collected at 3 h for mRNA isolation. *WRKY38* transcript abundance was measured via reverse transcription quantitative PCR (RT-qPCR) and relative expression ($2^{-\Delta Ct}$) for each condition was determined relative to *UBC21* abundance. Box and whisker dot plot represents the median (center line), first and third quartiles (box edges), and the minimum and maximum values (whiskers). One biological replicate consists of 15-18 pooled seedlings (n = 2-3).

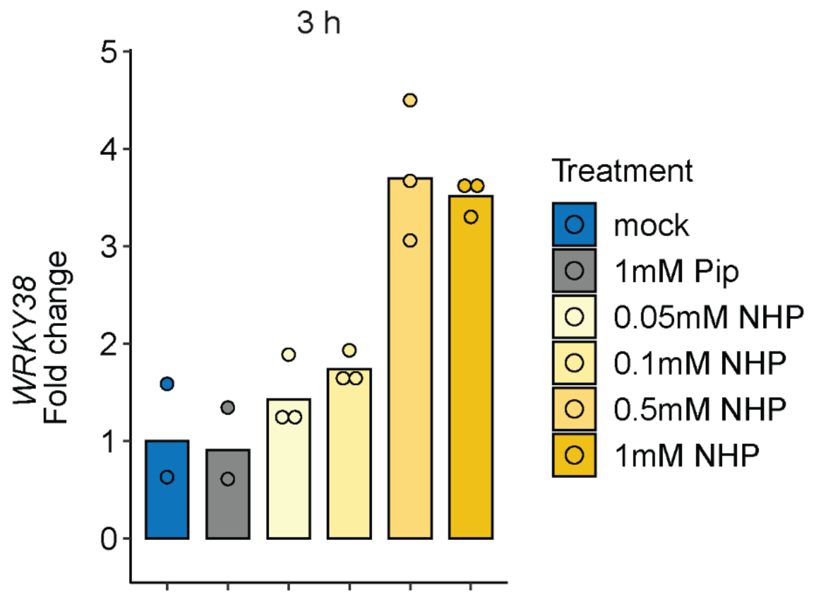

Supplementary Figure S2 Changes in *WRKY38* mRNA abundance at 3h in response to NHP concentrations.

Hydroponically grown wild type (Col-0) Arabidopsis seedlings were treated with mock, 1 mM Pip, and a range of NHP concentrations (0.05 mM, 0.1 mM, 0.5 mM, and 1 mM) at 10 dpg. After treatment, seedlings were collected at 3 h for mRNA isolation. *WRKY38* transcript abundance was first normalized relative to *UBC21* expression then fold change determined relative to mock ($2^{-\Delta\Delta Ct}$). The data for mock, 1 mM Pip, and 1 mM NHP samples are the same as shown in Supplementary Figure S1. One biological replicate consists of 15-18 pooled seedlings (n = 2-3).

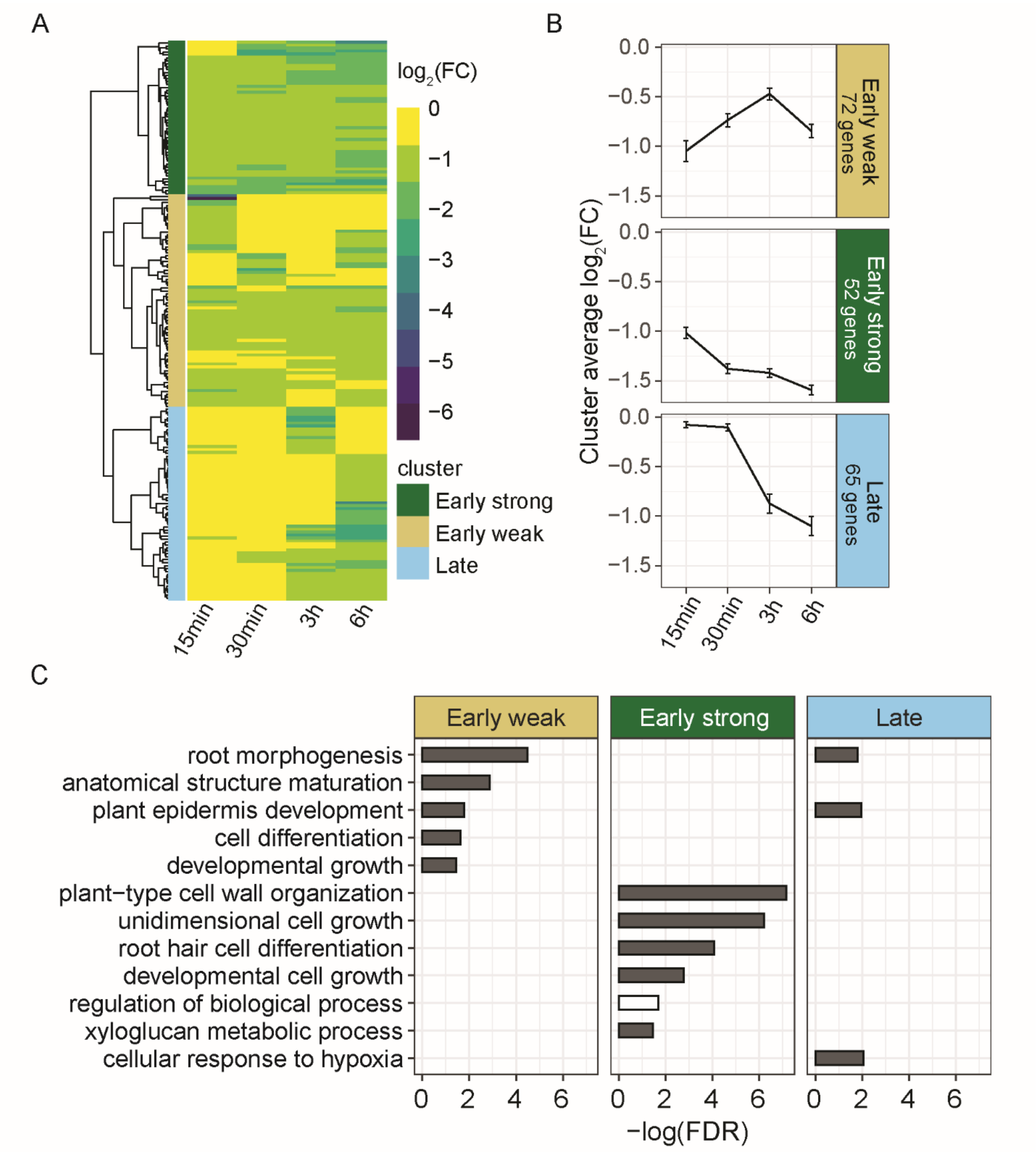

Supplementary Figure S3 Profile of early NHP-downregulated genes in wild type seedlings.

Three biological replicates of 15 pooled seedlings were performed for each timepoint and genotype. The transcriptome of the treated seedlings was analyzed via RNA-seq and log_2(fold change (FC)) for each time point determined relative to mock treated samples,

values were considered significant with a $P_{adj} < 0.05$. A, Heatmap of genes downregulated ($log_2(FC) < -1$, $P_{adj} < 0.05$) in response to NHP treatment in wild type seedlings. Hierarchical clustering was applied to the Euclidean distances determined from the $log_2(FC)$ values across the indicated time points, resulting in three distinct clusters of gene expression. B, Average $log_2(FC)$ of all genes within each cluster defined in (A) showing the expression trends across the four time points. Error bars represent the standard error of the mean (SEM). C, Biological processes of significantly overrepresented (gray bar) or underrepresented (white bar) (FDR < 0.05) Gene Ontology (GO) terms for the genes within each cluster are shown. Bars represent the negative log(FDR) of each significantly enriched GO term. Bars were omitted if the term was not significantly over- or underrepresented for the defined cluster.

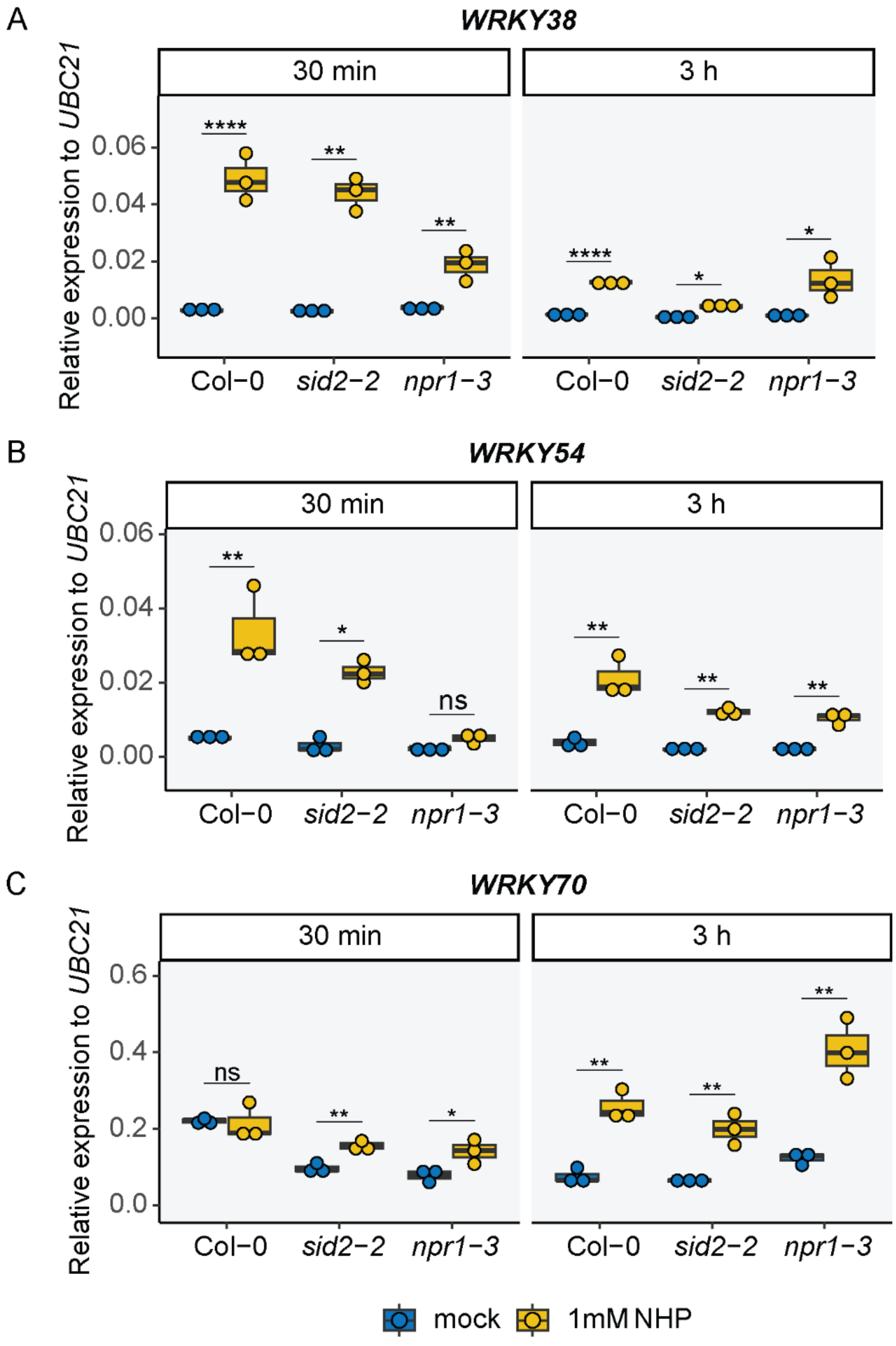

Supplementary Figure S4 Expression of select early strong cluster *WRKY* transcripts in *sid2-2* and *npr1-3* seedlings.

Hydroponically grown wild type (Col-0), *sid2-2*, and *npr1-3* Arabidopsis seedlings were treated at 10 dpg with mock or 1 mM NHP then collected after 30 min and 3 h for mRNA isolation. Transcript abundance was measured via RT-qPCR for *WRKY38* (A), *WRKY54*

(B), and *WRKY70* (C). Relative expression ($2^{-\Delta Ct}$) for each condition was determined relative to *UBC21* abundance. Box and whisker dot plot represents the median (center line), first and third quartiles (box edges), and the minimum and maximum values (whiskers) of three biological replicates from 15-18 pooled seedlings. Asterisks indicate a significant difference between treatment at each time point (two-tailed t-test; *P < 0.05, **P < 0.01, ***P < 0.001, ****P < 0.0001, ns = not significant).

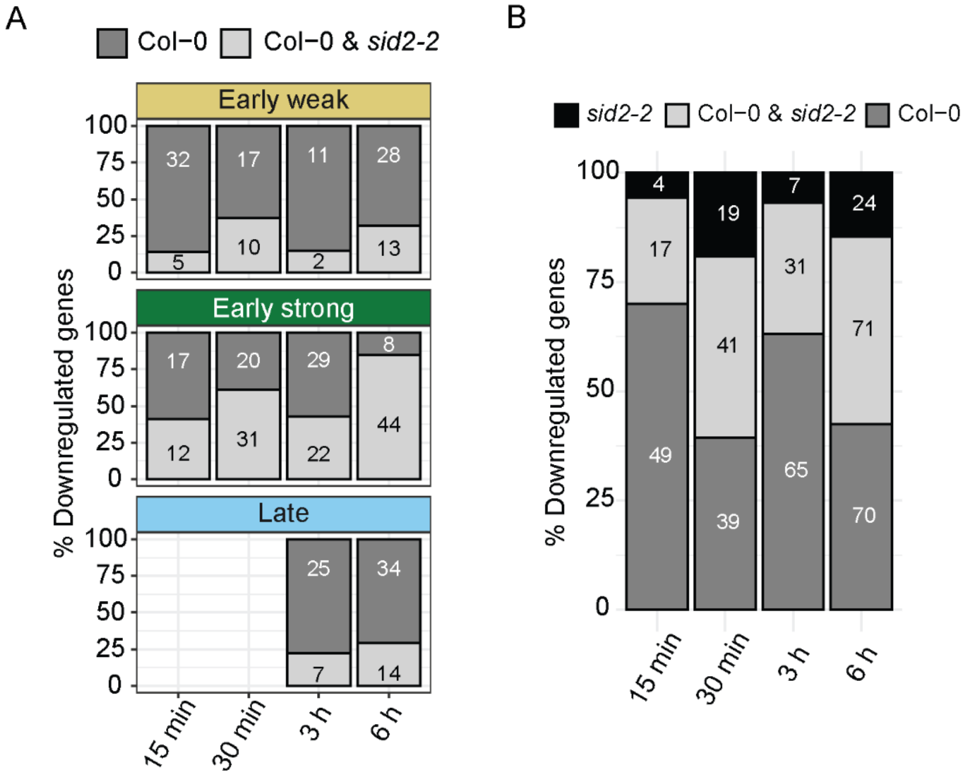

Supplementary Figure S5 Comparison of NHP downregulated genes in wild type and *sid2-2*.

A, Percent of NHP downregulated genes expressed in wild type (Col-0, SA-dependent) and in both wild type and the *sid2-2* mutant (SA-independent) for the wild type expression clusters defined in Supplementary Figure S3. Total number of genes in each condition is indicated. B, Total number of all NHP downregulated genes ($\log_2$(FC) < -1, P_{adj} < 0.05) per time point unique to wild type (Col-0), shared between wild type and *sid2-2*, and unique to *sid2-2*. The number of downregulated genes in each grouping is indicated.

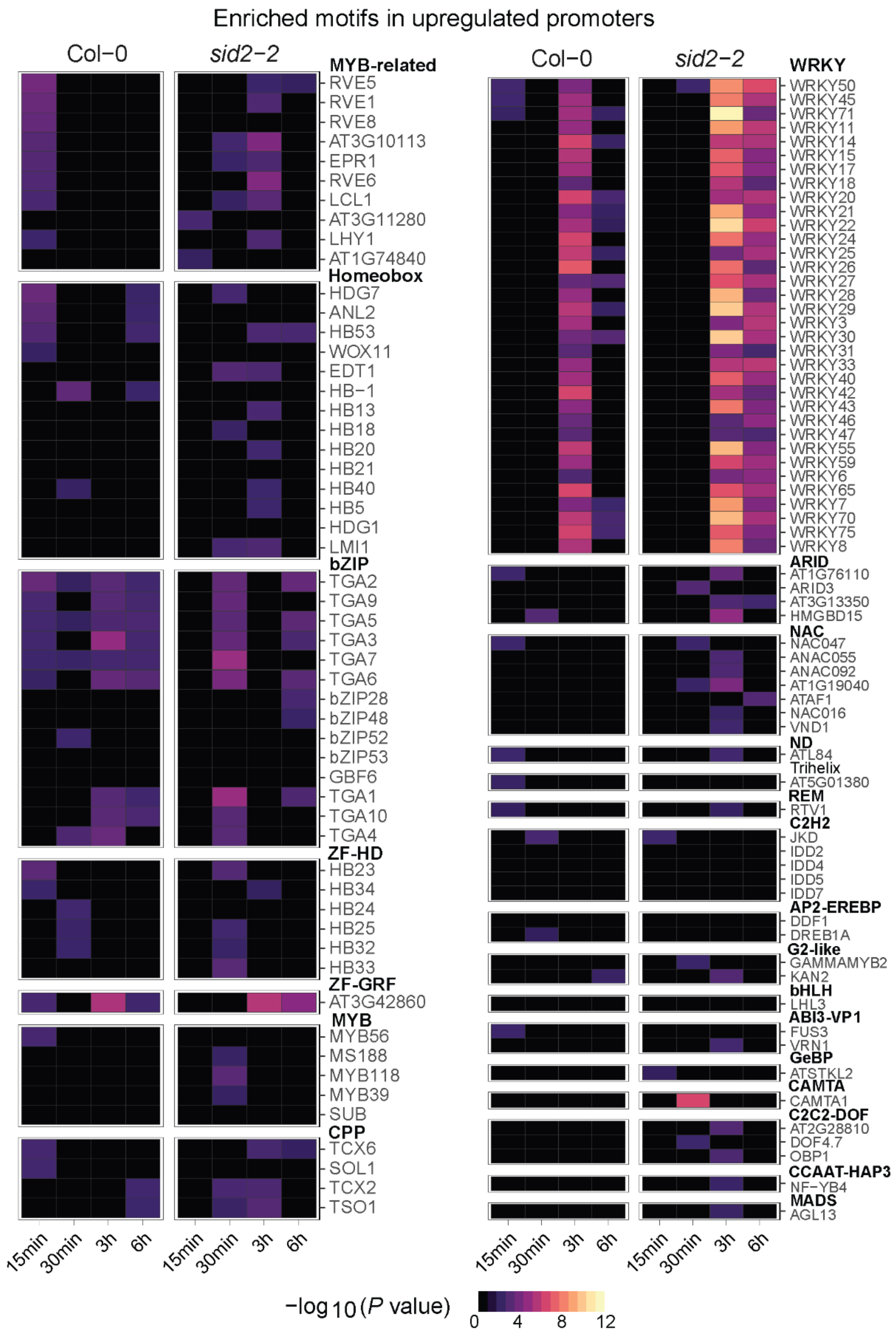

Supplementary Figure S6 All *cis*-regulatory elements (CREs) in the promoters of NHP-upregulated genes in wild type (Col-0) and *sid2-2* seedlings.

All genes with increased transcript abundance upon NHP treatment (log_2(FC) > 0 and P_{adj} < 0.05) were grouped by time point for the indicated genotype. The promoters (-1000 bp upstream of the transcriptional start site) of each grouping were analyzed for the presence of TF-binding motifs (CREs) from the DAPseq database (O'Malley et al., 2016). Enrichment of these CREs was determined relative to TF-binding motifs in promoters pulled from a random sampling of genes detected in this RNA-seq experiment. The -log_2(*P* value) of the enrichment analysis is indicated by the scale bar with black indicating no significant enrichment. The TF family name is indicated in bold above each group and the TF name of the identified binding motif is listed underneath.

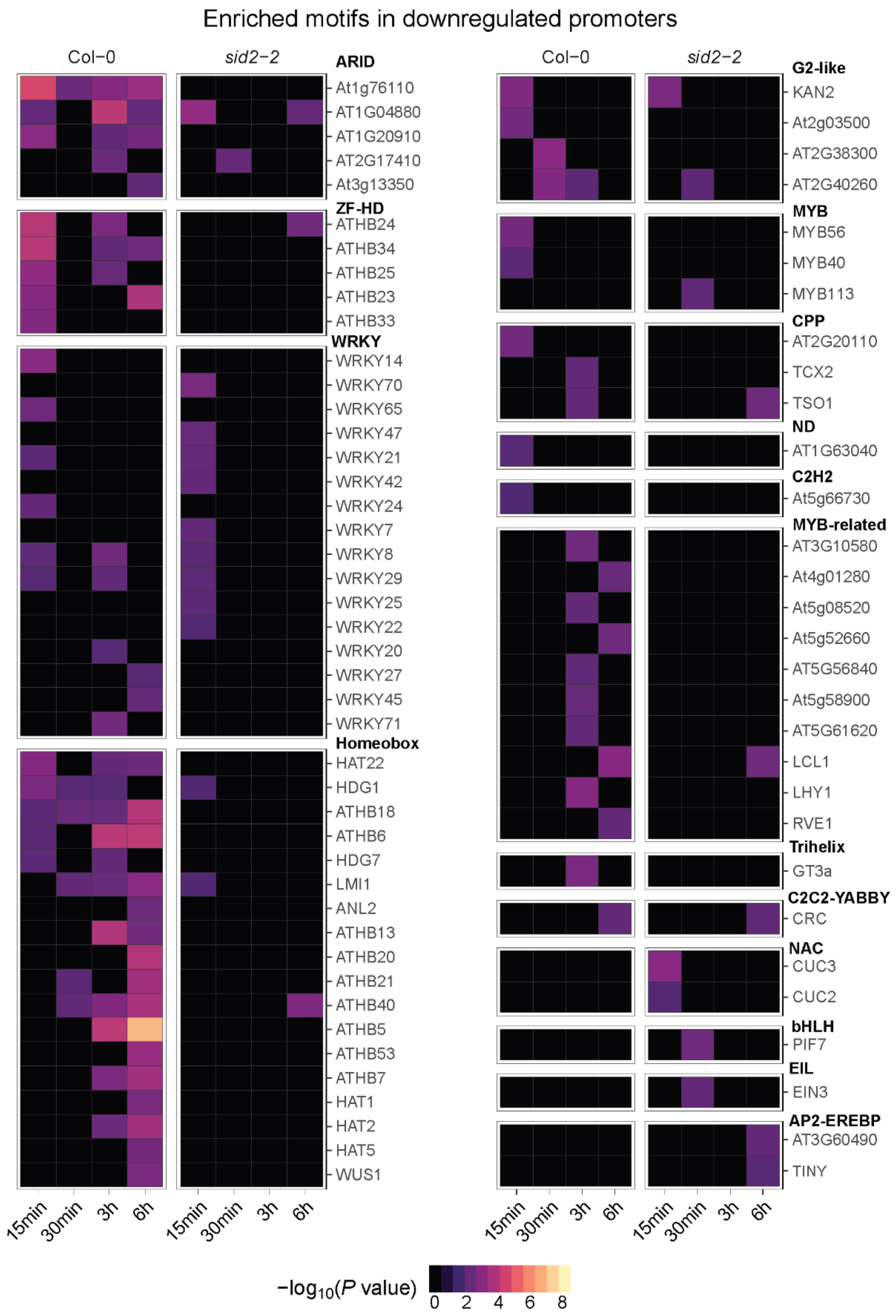

Supplementary Figure S7 All *cis*-regulatory elements (CREs) in the promoters of NHP-downregulated genes in wild type (Col-0) and *sid2-2* seedlings.

All genes with decreased transcript abundance upon NHP treatment (log_2(FC) < 0 and P_{adj} < 0.05) were grouped by time point for the indicated genotype. The promoters (-1000 bp upstream of the transcriptional start site) of each grouping were analyzed for TF-binding motifs (CREs) from the DAPseq database (O'Malley et al., 2016). Enrichment of these CREs was determined relative to TF-binding motifs of promoters pulled from a random sampling of genes detected in this RNA-seq experiment. The -log_2(P value) of the enrichment analysis is indicated by the scale bar with black indicating no significant enrichment. The TF family name is indicated in bold above each group and the TF name of the identified binding motif is listed underneath.

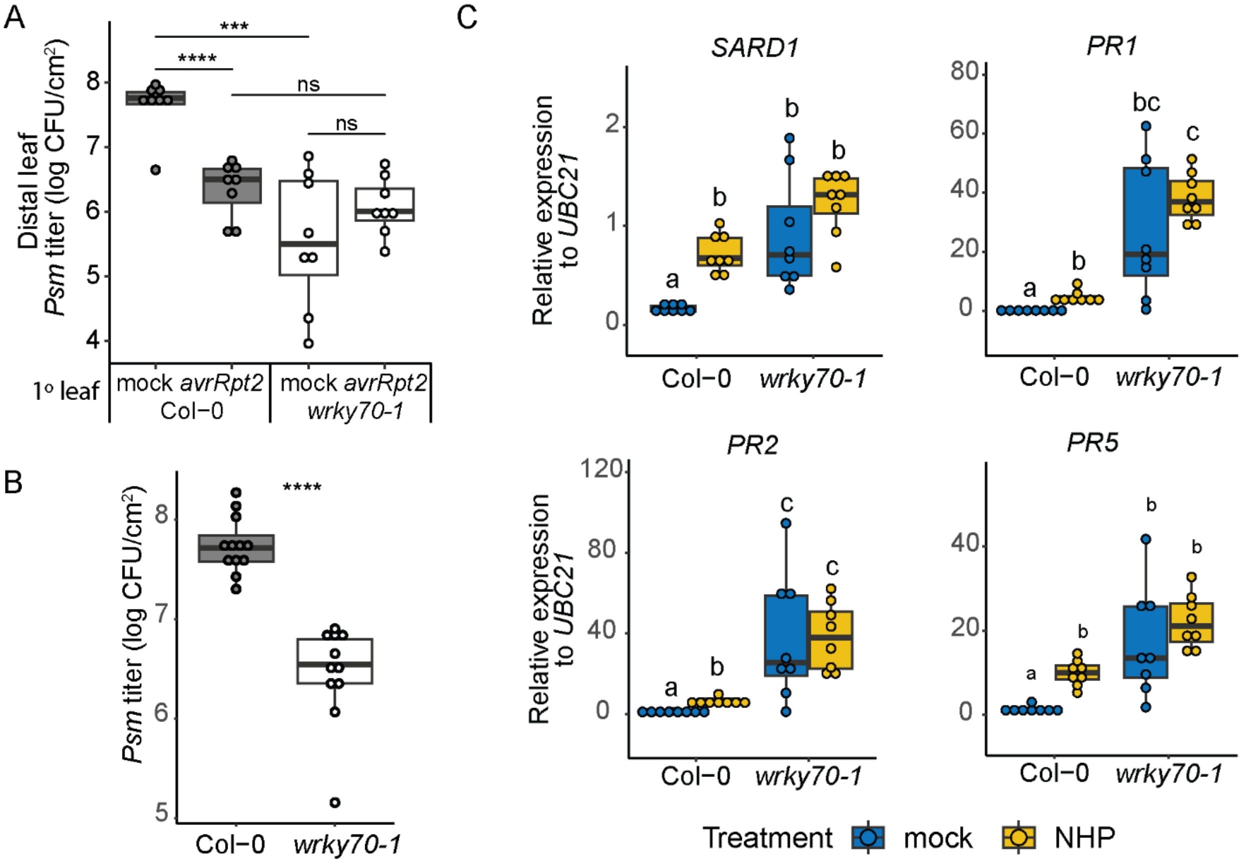

Supplementary Figure S8 Loss of *WRKY70* results in enhanced resistance to *Psm* infection and elevated levels of defense transcripts independent of NHP treatment.

A, Bacterial growth in the distal leaves of mock and avirulent pathogen treated wild type and *wrky70-1* mutant plants. Three lower (1°) leaves of 4.5-week-old plants were infiltrated with 10 mM $MgCl_2$ (mock) or 5×10^6 CFU/mL of avirulent *Pst avrRpt2*, one distal leaf was inoculated 2 d later with *Psm*, followed by quantification of *Psm* growth 3 dpi (n = 8). B, Resistance to *Psm* in wild type (Col-0) and *wrky70-1* plants without priming treatment. Two leaves of wild type and *wrky70-1* mutant plants were inoculated with a 1 x 10^5 CFU/ mL suspension of *Psm*, 3 dpi the two leaves were pooled, and bacterial titer measured (n = 11-12). Asterisks indicate significant differences in bacterial titer for panels A and B (two-tailed t-test; **P* < 0.05, ***P* < 0.01, ****P* < 0.001, *****P* < 0.0001, ns = not significant). C, Expression of defense marker genes *SARD1*, *PR1*, *PR2*, and *PR5*. Three lower leaves of 4.5-week-old plants were infiltrated with water or 1 mM NHP. Treated leaves were then collected at 24 h for mRNA isolation and transcript abundance was

determined relative to *UBC21* for each condition ($2^{-\Delta Ct}$). Data is pooled from two separate experiments. Statistical analysis was performed using a one-way ANOVA and post hoc Sidak test, different letters indicated statistical differences between means with $P < 0.05$, n = 8. All box and whisker dot plot represents the median (center line), first and third quartiles (box edges), and the minimum and maximum values (whiskers).

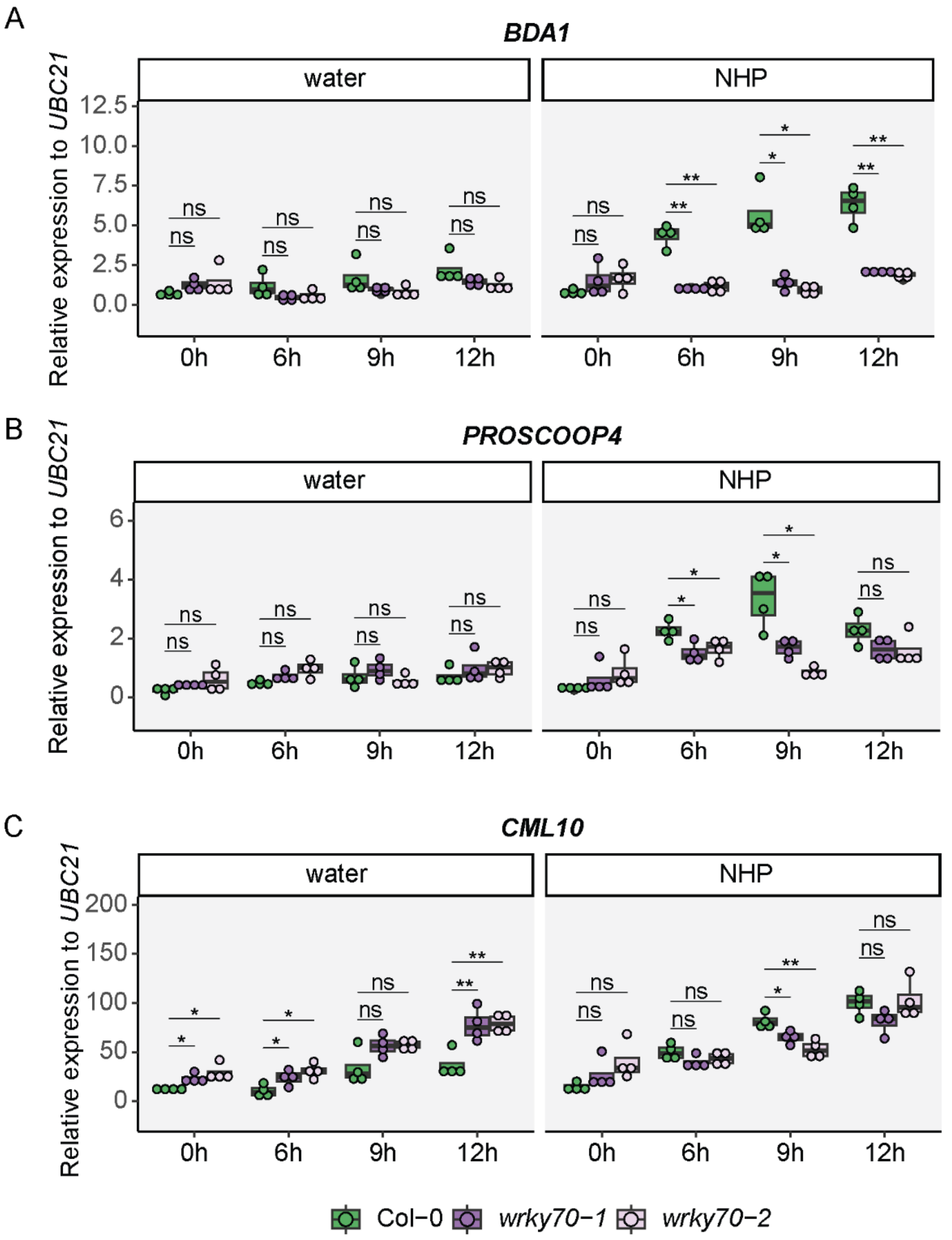

Supplementary Figure S9 Expression of NHP-responsive transcripts in *wrky70-1* and *wrky70-2* plants.

Expression of *BDA1* (A), *PROSCOOP4* (B), and *CML10* (C) in *wrky70* mutant lines. Three leaves of 4.5-week-old wild type (Col-0), *wrky70-1*, and *wrky70*-2 mutant plants were infiltrated with water or 1 mM NHP. Samples collected at 0 h were untreated before collection. Transcript abundance was determined relative to that of *UBC21* for each

condition ($2^{-\Delta Ct}$). Box and whisker dot plot represents the median (center line), first and third quartiles (box edges), and the minimum and maximum values (whiskers) of four biological replicates. Asterisks indicate a significant difference between wild type and *wrky70-1* or *wrky70-2* at each time point (two-tailed t-test; *P < 0.05, **P < 0.01, ns = not significant). The data for wild type and *wrky70-1* are the same as shown in Figure 6.

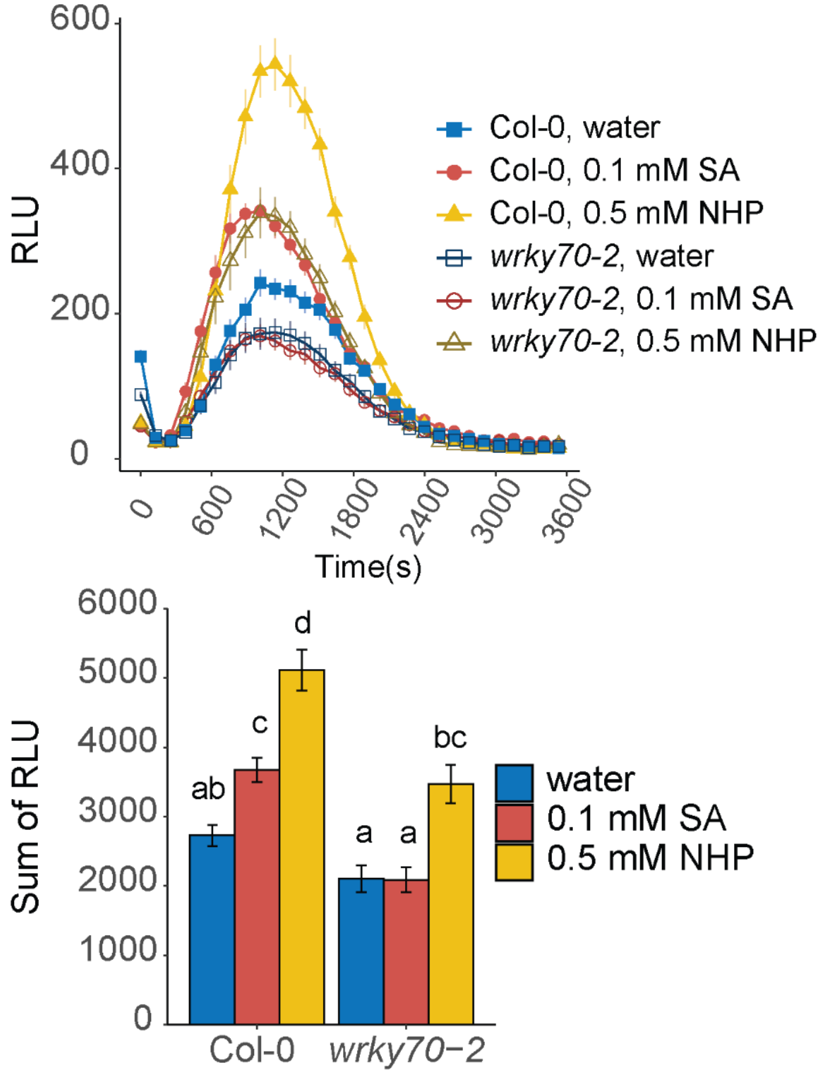

Supplementary Figure S10 NHP enhanced ROS production is compromised in *wrky70-2* plants.

Flg22-elicited reactive oxygen species (ROS) quantification in *wrky70-2* plants. Four leaf discs from 5 to 6-week-old wild type and *wrky70-2* plants were pretreated by floating leaf discs on water, 0.1 mM salicylic acid (SA), or 0.5 mM *N*-hydroxy-pipecolic acid (NHP) for 24 h before treating with 100 nM flg22 peptide in horseradish peroxidase and Luminol. Top, traces of the average relative luminescence units (RLU) over the indicated run time. Each point represents the average of leaf discs from 6 plants (n = 24) from two experiments and error bars represent the standard error of the mean (SEM). Bottom, total quantification of RLUs from top plot. For each leaf disc, RLUs from all time point were summed by condition over the run time and averaged with error bars representing the

SEM (n = 24). Statistical analysis was performed using a one-way ANOVA and post hoc Sidak test, different letters indicated statistical differences between means with $P < 0.05$.

Supplementary Table S3. Primers used in this study.

Description	Name	Sequence (5' - 3')	Reference
**RT-qPCR analysis**			
UBC21	qFW	TCAAATGGACCGCTCTTATC	Sigma-Aldrich
	qRV	CACAGACTGAAGCGTCCAAG	
FMO1	qFW	TCTTCTGCGTGCCGTAGTTTC	Návarová et al., 2012
	qRV	CGCCATTTGACAAGAAGCATAG	
ICS1	qFW	GCAAGAGTGCAACATCTATATTCTC	Bernsdorff et al., 2016
	qRV	CACAAACAGCTGGAGTTGGA	
UGT76B1	qFW	TCCCGAGGATTGTTCTCCGAAC	this study
	qRV	AACCGGTGAGTCTGCCTTAGTC	
WRKY38	qFW	GCCCCTCCAAGAAAAGAAAG	this study
	qRV	CCTCCAAAGATACCCGTCGT	
WRKY54	qFW	CTTCATGTGGGGATGATTCGGCG	this study
	qRV	TGTGATCTCGTCTTTCTAGTGTAGCA	
WRKY70	qFW	GAGATCGGAGACGTGTACTATAGAGTCG	Liu et al., 2021
	qRV	GCACCCTTGGGTATACTTGTGTGTG	
SARD1	qFW	CCTCAACCAGCCCTACGTTA	Truman et al., 2013
	qRV	TAGTGGCTCGCAGCATATTG	
PR1	qFW	GTGCTCTTGTTCTTCCCTCG	Návarová et al., 2012
	qRV	GCCTGGTTGTGAACCCTTAG	
PR2	qFW	TCAAGGAAGGTTCAGGGATG	Chen et al., 2018
	qRV	TCGAGATTTGCGTCGAATAG	
PR5	qFW	AAGGTCATGGATCAGAACAA	Chen et al., 2018
	qRV	GCAGAAAGTGATTTCGTAGT	
BDA1	qFW	ACGAGCCAGAGGATCTCACATG	this study
	qRV	ACTCCTGTAACGTGCAATTCGC	
PROSCOOP4	qFW	CAGGCCAAACGTCTCCAACAAA	this study
	qRV	GCGATAGGAGGTTTGACGATGC	
CML10	qFW	CTCCGGTGGAAGCAAACTGATG	this study
	qRV	GCGCTGCGATGATATTTTTGGC	
**Genotype analysis**			
SALK T-DNA border	LBb1.3	ATTTTGCCGATTTCGGAAC	
SALK_017254	LP	AACCAGTACCACATCGATGAGAC	
	RP	GCTGGTGTTGTTCTCTTGCTC	
SALK_025198 (*wrky70-1*)	LP	TGATCTTCGGAATCCATGAAG	
	RP	CAAACCACACCAAGAGGAAAG	
SALK_022198	LP	TGAATATCTCTCAAAACCCTAGCC	
	RP	TTGCTTTCAAACCATGCTTTG	
SALK_039436	LP	GCACGAAAGTAGCCATGAAAG	
	RP	TGATTTCTGCAATGAATAAATGTG	
GABI kat T-DNA border	8474_DR35	ATAATAACGCTGCGGACATCTACATTTT	
GABI_324D11 (*wrky70-2*)	LP	CAAACCACACCAAGAGGAAAG	
	RP	ATGACAAGTCATTCTCCGTGG	
pDs-Lox-LT6 T-DNA border	LT6	AATAGCCTTTACTTGAGTTGGCGTAAAAG	
WiscDsLox489-492C21	LP	ATTTGGTAAACCCAAATTGGC	
	RP	CGATGAAGGAGGATAAGAGCC	
dSpm T-DNA border	Spm32	TACGAATAAGAGCGTCCATTTTAGAGTGA	

SM_3_38820	LP	TTACGCGACGACATCAAATAG	
	RP	CATAAGCTTGATGAAGCTGGC	
sid2-2	FW	TGTCTGCAGTGAAGCTTTGG	Bernacki et al., 2021
	RV	CGAAGAAATGAAGAGCTTGGA	
npr1-3	FW	GGGATATACGGTGCTTCATGTT	DeFraia et al., 2010
	RV	CTTTACACCCGGTGATGTTCT	

Supplementary Table S4. Setup of early NHP RNA-sequencing experiment.

**Samples**	**Treatment**	**Timepoint (min)**	**Genotype**	**Rep**	**Batch**	**Group**	**Label**
JF.01	mock	15	Col-0	r1	1	col_15_mock	JF.01_col_15_mock_r1
JF.02	mock	15	Col-0	r2	1	col_15_mock	JF.02_col_15_mock_r2
JF.03	mock	15	Col-0	r3	1	col_15_mock	JF.03_col_15_mock_r3
JF.10	NHP	15	Col-0	r1	2	col_15_NHP	JF.10_col_15_NHP_r1
JF.11	NHP	15	Col-0	r2	2	col_15_NHP	JF.11_col_15_NHP_r2
JF.12	NHP	15	Col-0	r3	2	col_15_NHP	JF.12_col_15_NHP_r3
JF.19	mock	30	Col-0	r1	3	col_30_mock	JF.19_col_30_mock_r1
JF.20	mock	30	Col-0	r2	3	col_30_mock	JF.20_col_30_mock_r2
JF.21	mock	30	Col-0	r3	3	col_30_mock	JF.21_col_30_mock_r3
JF.28	NHP	30	Col-0	r1	4	col_30_NHP	JF.28_col_30_NHP_r1
JF.29	NHP	30	Col-0	r2	4	col_30_NHP	JF.29_col_30_NHP_r2
JF.30	NHP	30	Col-0	r3	4	col_30_NHP	JF.30_col_30_NHP_r3
JF.37	mock	180	Col-0	r1	5	col_180_mock	JF.37_col_180_mock_r1
JF.38	mock	180	Col-0	r2	5	col_180_mock	JF.38_col_180_mock_r2
JF.39	mock	180	Col-0	r3	5	col_180_mock	JF.39_col_180_mock_r3
JF.46	NHP	180	Col-0	r1	6	col_180_NHP	JF.46_col_180_NHP_r1
JF.47	NHP	180	Col-0	r2	6	col_180_NHP	JF.47_col_180_NHP_r2
JF.48	NHP	180	Col-0	r3	6	col_180_NHP	JF.48_col_180_NHP_r3
JF.55	mock	360	Col-0	r1	7	col_360_mock	JF.55_col_360_mock_r1
JF.56	mock	360	Col-0	r2	7	col_360_mock	JF.56_col_360_mock_r2
JF.57	mock	360	Col-0	r3	8	col_360_mock	JF.57_col_360_mock_r3
JF.64	NHP	360	Col-0	r1	8	col_360_NHP	JF.64_col_360_NHP_r1
JF.65	NHP	360	Col-0	r2	9	col_360_NHP	JF.65_col_360_NHP_r2
JF.66	NHP	360	Col-0	r3	9	col_360_NHP	JF.66_col_360_NHP_r3
JF.07	mock	15	sid2-2	r1	1	sid2_15_mock	JF.07_sid2_15_mock_r1
JF.08	mock	15	sid2-2	r2	1	sid2_15_mock	JF.08_sid2_15_mock_r2
JF.09	mock	15	sid2-2	r3	1	sid2_15_mock	JF.09_sid2_15_mock_r3
JF.16	NHP	15	sid2-2	r1	2	sid2_15_NHP	JF.16_sid2_15_NHP_r1
JF.17	NHP	15	sid2-2	r2	3	sid2_15_NHP	JF.17_sid2_15_NHP_r2
JF.18	NHP	15	sid2-2	r3	3	sid2_15_NHP	JF.18_sid2_15_NHP_r3
JF.25	mock	30	sid2-2	r1	4	sid2_30_mock	JF.25_sid2_30_mock_r1
JF.26	mock	30	sid2-2	r2	4	sid2_30_mock	JF.26_sid2_30_mock_r2
JF.27	mock	30	sid2-2	r3	4	sid2_30_mock	JF.27_sid2_30_mock_r3
JF.34	NHP	30	sid2-2	r1	5	sid2_30_NHP	JF.34_sid2_30_NHP_r1
JF.35	NHP	30	sid2-2	r2	5	sid2_30_NHP	JF.35_sid2_30_NHP_r2

JF.36	NHP	30	sid2-2	r3	5	sid2_30_NHP	JF.36_sid2_30_NHP_r3
JF.43	mock	180	sid2-2	r1	6	sid2_180_mock	JF.43_sid2_180_mock_r1
JF.44	mock	180	sid2-2	r2	6	sid2_180_mock	JF.44_sid2_180_mock_r2
JF.45	mock	180	sid2-2	r3	6	sid2_180_mock	JF.45_sid2_180_mock_r3
JF.52	NHP	180	sid2-2	r1	7	sid2_180_NHP	JF.52_sid2_180_NHP_r1
JF.53	NHP	180	sid2-2	r2	7	sid2_180_NHP	JF.53_sid2_180_NHP_r2
JF.54	NHP	180	sid2-2	r3	7	sid2_180_NHP	JF.54_sid2_180_NHP_r3
JF.61	mock	360	sid2-2	r1	8	sid2_360_mock	JF.61_sid2_360_mock_r1
JF.62	mock	360	sid2-2	r2	8	sid2_360_mock	JF.62_sid2_360_mock_r2
JF.63	mock	360	sid2-2	r3	8	sid2_360_mock	JF.63_sid2_360_mock_r3
JF.70	NHP	360	sid2-2	r1	9	sid2_360_NHP	JF.70_sid2_360_NHP_r1
JF.71	NHP	360	sid2-2	r2	9	sid2_360_NHP	JF.71_sid2_360_NHP_r2
JF.72	NHP	360	sid2-2	r3	9	sid2_360_NHP	JF.72_sid2_360_NHP_r3

Supplemental Table S1 Full RNA-seq dataset of the early transcriptional response to exogenous NHP in Col-0 and *sid2-2*.

Supplemental Table S2 Full set of $\log_2$ fold change values grouped by upregulated and downregulated wild type gene clusters.

Supplemental Table S3 Primer sequences used in this study.

Supplemental Table S4 Setup of early NHP RNA-sequencing experiment.
