# Supplementary material for: Transcriptome analysis reveals role of transcription factor WRKY70 in early N-hydroxy-pipecolic acid signaling
Source: Plant Physiol. 2024 Oct 15;197(1):kiae544. doi: 10.1093/plphys/kiae544 (PMC11663584; doi:10.1093/plphys/kiae544)
Supplement: kiae544_Supplementary_Data [file pp.00594.2024-s06.pdf › pp.00594.2024-s06.pdf]

Plant Physiology®

Transcriptome analysis reveals role for WRKY70 in early N-hydroxy-pipecolic acid signaling

Jessica Foret, Jung-Gun Kim, Elizabeth Sattely, and Mary Beth Mudgett

Review Timeline:

Submission Date:	23-Apr-2024
Editorial Decision:	04-Jun-2024
Revision Received:	08-Jul-2024
Accepted:	31-Jul-2024

04-Jun-2024

Prof. Mary Beth Mudgett
Stanford University
Department of Biological Sciences
Stanford, California 94305

RE: Transcriptome analysis reveals role for WRKY70 in early N-hydroxy-pipecolic acid signaling

Dear Dr. Mudgett:

We have received reviews of your manuscript, which you may view at the end of this letter. Both reviewers were generally supportive of your work, revealing novel insights in the early and later-acting signalling steps underpinning NPH-dependent SAR. However, as you will see, both reviewers raised various pionts and suggestions that require your attention. Upon further consulation with both reviewers, we agreed that your manuscript may be accetable for publicaiton in Plant Physiology, provided you are able to address the major and minor concerns raised.

Reviewer #1 raised some valid points about the SA- and NPR1-dependent expression of early transient genes by NPH. We also noted that these SID2-dependent genes appear to be enriched with jasmonic acid -related GO terms, rather than SA-related GO terms (Fig. 3), which may be relevant to discuss in context of earlier observations that JA acts as a signal for early SAR development (i.e. doi: 10.1073/pnas.0605423104) As further alluded by reviewer #1, I believe it would be good if you could explain in more detail the negative and positive actions of WRKY70 on SA-dependent resistance and ABA/ROS-dependent defences, respectively, suggesting a possible role of WRKY70 in the fine-tuning and signalling cross-talk between SA/JA/ABA-dependent defences. In this context, a graphical model of the main conclusions would be helpful.

Reviewer #2 made a further valid comment as to why only WRKY70 was singled out for further analysis? Although you screened six wrky mutants for SAR responses to NPH (of which wrky62 also showed am interesting mutant phenotype, which was not further discussed), we were particularly concerned by the fact that these results are mostly based on single T-DNA mutant alleles. Only some analyses (gene expression/ROS) were repeated and validated in an independent T-DNA insertion mutant (wkry70-2) but this does not include quantification of SAR to NPH treatment. Accordingly, I believe it is important to validate these mutant phenotypes in independent mutants.

When you are ready to submit your revision, click the below link and find the red arrow under your Author Tasks heading for this revision submission.

If you want to visit this version of the manuscript, you may click the link below and then click on the folder that reads "Post Decision Manuscripts".

Link Not Available

When uploading the revised version of this article, please be sure to include as complete an answer as possible to the referees' criticisms during the first round of reviews in the "response to reviewers" section.

Plant Physiology considers a revision for up to 60 days after a manuscript has been provisionally accepted; limited extension of this timeframe is at the discretion of the Monitoring Editor. A reminder notice will be sent to you 2 weeks before the deadline, at which time you may choose to contact the editorial office to request an extension; if so, please provide a justification for your request. After the expiration of this deadline, the article would need to be submitted as a resubmission and would be handled as such.

Thank you very much for giving us an opportunity to review this work. I look forward to receiving the next version.

Sincerely,

Editor
Plant Physiology

Want to add this revision deadline to your calendar? Click below!

-- Reviewer comments:
Reviewer #1 (Comments for the Author):

This manuscript describes a study focusing on NHP-induced early transcriptional changes that has not be reported previously. The authors found that at least two waves of transcription within minutes to hours after NHP treatment. The first wave of transcription includes WRKY transcription factors (TFs) and the second wave WRKY-regulated defense genes. Moreover, the authors revealed that most of the genes induced by NHP in minutes are SA-dependent, whereas those within hours are SA-independent. Finally, the authors show that the first wave gene WRKY70, which is a well-documented negative regulator plays a positive role in the NHP-induced second wave transcription and flg22-induced ROS accumulation. This manuscript provides useful information about NHP-induced transcriptional changes, which is valuable for understanding NHP signaling and SAR.

It has been well documented in many biological systems that transcriptional cascades are mediated by TFs. It is intriguing that NHP induces TFs (mostly WRKYs) only in the first wave (not in the second wave), suggesting that later transcriptional changes are mainly mediated by WRKYs and/or constitutively expressed TFs. Indeed, TGA factors have been shown to play a definitive role in NHP signaling. The authors mentioned TGA factors in Discussion; it would be very informative to determine if the first wave of WRKY transcription is controlled by TGAs (e.g., NHP-induced expression of the WRKYs at 15 or 30 min in tga2/3/5/6?).

The early transient genes are mainly sid2 dependent. It is not clear if their transcription requires basal SA or new SA biosynthesis. Furthermore, are the early strong sid2-independent genes (e. g., WRKYs) NPR1 dependent at the early time points?

Based on the transcriptome data, the authors focus on the positive role of WRKY70 in NHP signaling. It is known that some WRKYs are positive immune regulators and others are negative regulators. NHP activates both positive and negative TFs. The main role of the negative TFs, such as WRKY70 and WRKY 62, would be to balance or shut off the transcription at the later stage. This should be tested or at least discussed in the manuscript.

In Fig. S1, can the authors explain why Pip did not induce WRKY38?

In Fig. S3, the white bar represents depleted biological processes. Can the authors clarify "depleted"?

In Fig. S7 legend, "D" should be "C".

Reviewer #2 (Comments for the Author):

The study investigated the role of WRKY 70 in N-hydroxypipecolic acid (NHP) associated systemic acquired resistance (SAR) and its relationship with salicylic acid (SA) in SAR in Arabidopsis, especially at the early infection stage.

Strengths:
1. The study provided a detailed examination of the transcriptional responses to NHP over various time points, highlighting both early and late gene expression changes.
2. The use of the sid2-2 mutant (defective in SA biosynthesis) allows for the dissection of the relationship between NHP and SA, showing that NHP can induce gene expression changes independent of SA.
3. The study identified the key transcription factors (TFs) like TGA and WRKY that are involved in NHP signaling, enriching our understanding of the molecular mechanisms underlying SAR.
4. The research included functional validation by examining the role of specific WRKY TFs (e.g., WRKY70) in SAR, providing insights into the secondary transcriptional response driven by NHP.
5. The combination of gene expression analysis, TF binding site enrichment, and mutant phenotype characterization enhances the robustness of the findings.

Weaknesses:
1. While the study identified several WRKY TFs involved in NHP signaling, only WRKY70 is thoroughly investigated. Other TFs and their roles in NHP-induced SAR could be explored further or at least show some future insights.
2. The findings sometimes reiterated known interactions between SA and JA signaling pathways, however, it needs to be further discussed how they function in SAR associated with WRKY70 and NHP.
3. Although the study covered multiple time points, the resolution between early and late responses could be improved to better understand the dynamics of NHP signaling.
4. The study identified the correlations between TF binding sites and gene expression changes but did not delve deeply into the mechanistic details of how NHP influences TF activity and gene expression.

5. Method parts can be further improved by providing more details, such as the chemical compound preparation and related place for the purchase.
6. It will be much helpful to provide a summary figure to show the related mechanisms and function of WRKY in NHP related SAR.

Overall, the study makes significant contributions to understanding the role of NHP in plant immunity, particularly in relation to SA and WRKY TFs at early stage, but could be strengthened b

Stanford University
Department of Biology

Professor Mary Beth Mudgett, PhD
Susan B. Ford Professor
Stanford Friends University Fellow in Undergraduate Education
Senior Associate Dean for Natural Sciences, School H&S

PP2024-RA-00594 - *Transcriptome analysis reveals role for WRKY70 in early N-hydroxy-pipecolic acid signaling* by Foret, Kim, Sattely and Mudgett.

MAJOR CHANGES:
Below is a summary of the major changes made to the manuscript based on the comments by the editor and referees. We have included a revised manuscript with tracked changes to view all changes. The major changes include:

- Changed use of "low SA" to more accurate "basal levels of SA".
- Clarified GO-term analysis by changing "depleted" to "underrepresented" and "enriched" to "overrepresented" for consistency and clarity.
- New data added – New Supplemental Figure S4: Measured transcript abundance of *WRKY38*, *WRKY54* and *WRKY70* in *npr1-3* and *sid2-2* to investigate dependence on NPR1 and ICS1/SID2.
- Renumbered Supplemental Figures:
    - Original S4 = new S5
    - Original S5 = new S6
    - Original S6 = new S7
    - Original S7 = new S8
    - Original S8 = new S9
    - Original S9 = new S10
- New data added – Figure 5E: Measured NHP-elicited SAR in wild type, *wrky70-1,* and *wrky70-2* following 0.5 mM NHP treatment. Reordered presentation of panels to be consistent with results presentation in the text.
- Added discussion of *wrky62* NHP-elicited SAR phenotype in results and discussion sections. Also noted why *wrky70* was selected for follow-up instead of *wrky62*.
- Clarified writing of results section "SA biosynthesis is dispensable for the early induction of a majority of NHP upregulated genes" to highlight which groups of genes are induced by NHP independent of SA.
- Added a new figure – Figure 8: Working Models
    - Summarizes early NHP upregulated expression and requirement of SA (A) and summarizes WRKY70 role in NHP signaling, ROS, and SAR (B).
    - Reference to model figure added throughout discussion section.
- Added discussion addressing role of WRKYs (WRKY62 and WRKY70) in negatively regulating NHP-elicited SAR.
- Added discussion of the putative role of WRKY70 in SA- and JA-responses induced by NHP signaling.

Department of Biology
371 Jane Stanford Way, 228A Gilbert Bioscience, Stanford, CA 94305-5020 / 1-650-723-3252 / mudgett@stanford.edu

- Added examples of genes to the ‘late strong’ results section.
- Added two new references: Cao et al., 1997 and AbuQamar et al., 2006.
- Further clarified chemical solution preparation in the methods section.

RESPONSE TO EDITOR & REVIEWERS:

Please note that in the response to the editor and reviewers, we reference the page and line numbers in the final version without tracked changes.

Editor Summary:
Reviewer #1 raised some valid points about the SA- and NPR1-dependent expression of early transient genes by NPH. We also noted that these SID2-dependent genes appear to be enriched with jasmonic acid -related GO terms, rather than SA-related GO terms (Fig. 3), which may be relevant to discuss in context of earlier observations that JA acts as a signal for early SAR development (i.e. doi: 10.1073/pnas.0605423104) As further alluded by reviewer #1, I believe it would be good if you could explain in more detail the negative and positive actions of WRKY70 on SA-dependent resistance and ABA/ROS-dependent defences, respectively, suggesting a possible role of WRKY70 in the fine-tuning and signalling cross-talk between SA/JA/ABA-dependent defences. In this context, a graphical model of the main conclusions would be helpful.

Response: We agree that it would be informative to comment on JA signaling, positive and negative actions of WRKY70 and provide a working model. We have included this information in the discussion section (page 24, lines 712-724) and also provide a new figure to summarize our working models (new Figure 8; page 54, lines 1264-1284).

Reviewer #2 made a further valid comment as to why only WRKY70 was singled out for further analysis? Although you screened six wrky mutants for SAR responses to NPH (of which wrky62 also showed am interesting mutant phenotype, which was not further discussed), we were particularly concerned by the fact that these results are mostly based on single T-DNA mutant alleles. Only some analyses (gene expression/ROS) were repeated and validated in an independent T-DNA insertion mutant (wkry70-2) but this does not include quantification of SAR to NPH treatment. Accordingly, I believe it is important to validate these mutant phenotypes in independent mutants.

Response: We agree that more work could be done to study other *wrky* alleles; however, given the time required to phenotype individual mutants, we prioritized pilot screens with a single allele and only followed up on candidates with strong phenotypes. The *wrky62* mutant phenotype is interesting and we noted this in the text, but chose not to study it further.

As requested, we characterized a second *wrky70 allele,* namely *wrky70-2.* We repeated the NHP induced SAR experiment and treated wild type, *wrky70-1*, and *wrky70-2* with mock or 0.5 mM NHP, followed by the *Psm* challenge. We found that both *wrky70* alleles exhibit an enhanced resistance phenotype and do not respond to NHP treatment, when compared to similarly treated wild type plants. These new data are presented in revised Figure 5E (page 50, lines 1226-1235) and discussed in the results section (page 16, lines 464-480).

Reviewer #1 (Comments for the Author):

This manuscript describes a study focusing on NHP-induced early transcriptional changes that has not be reported previously. The authors found that at least two waves of transcription within minutes to hours after NHP treatment. The first wave of transcription includes WRKY transcription factors (TFs) and the second wave WRKY-regulated defense genes. Moreover, the authors revealed that most of the genes induced by NHP in minutes are SA-dependent, whereas those within hours are SA-independent. Finally, the authors show that the first wave gene WRKY70, which is a well-documented negative regulator plays a positive role in the NHP-induced second wave transcription and flg22-induced ROS accumulation. This manuscript provides useful information about NHP-induced transcriptional changes, which is valuable for understanding NHP signaling and SAR.

It has been well documented in many biological systems that transcriptional cascades are mediated by TFs. It is intriguing that NHP induces TFs (mostly WRKYs) only in the first wave (not in the second wave), suggesting that later transcriptional changes are mainly mediated by WRKYs and/or constitutively expressed TFs. Indeed, TGA factors have been shown to play a definitive role in NHP signaling. The authors mentioned TGA factors in Discussion; it would be very informative to determine if the first wave of WRKY transcription is controlled by TGAs (e.g., NHP-induced expression of the WRKYs at 15 or 30 min in tga2/3/5/6?).

We agree that a better understanding of the regulation of *WRKY* transcription would be informative; however, we think this is beyond the scope of this initial study and will be an avenue for future research.

The early transient genes are mainly sid2 dependent. It is not clear if their transcription requires basal SA or new SA biosynthesis.

We revised the text to clarify that the expression of the early transient genes does not require new SA biosynthesis produced from ICS1/SID2. This is noted in several places within the text (page 2, line 35; page 5, line 135; page 12, line 335; page 20, lines 599-603; page 21, lines 631-634; page 54, lines 1267-1270).

Furthermore, are the early strong sid2-independent genes (e. g., WRKYs) NPR1 dependent at the early time points?

To address this question, we examined the mRNA abundance of three *WRKY* genes (*WRKY38*, *WRKY70*, and *WRKY54*) in Col-0 (wild type), *sid2-2*, and *nrp1-3* at 30 min and 3 hours post NHP treatment. We found that NHP significantly increased transcript abundance of *WRKY38* and *WRKY70* after 30 min and increased *WRKY38, WRKY54,* and *WRKY70* after 3 hours in *npr1-3* seedlings. Additionally, NHP increased transcript abundance of all three genes in *sid2-2* seedlings. Together, these data indicate that some WRKY TFs play a role in a SA- and NPR1-independent branch of early NHP signaling. These data are presented in the new Supplemental Figure S4, and described in the results (page 12, lines 340-349) and discussion (page 21, lines 626-634) sections.

Based on the transcriptome data, the authors focus on the positive role of WRKY70 in NHP signaling. It is known that some WRKYs are positive immune regulators and others are negative regulators. NHP activates both positive and negative TFs. The main role of the negative TFs, such as WRKY70 and WRKY 62, would be to balance or shut off the transcription at the later stage. This should be tested or at least discussed in the manuscript.

We have included discussion regarding the positive and negative functions of WRY70 in the discussion (page 22, lines 650-655; page 23, lines 687-699; page 24, lines 712-724) and in the working models figure (new Figure 8; page 54, lines 1264-1284).

In Fig. S1, can the authors explain why Pip did not induce WRKY38?

Our data indicates that NHP, and not Pip, increases *WRKY38* mRNA abundance at 3 hours post-treatment (Figure 1C and Figure S1). This suggests that Pip is not sufficient to induce this response. Data reported by Hartmann et al., 2018 indicate that Pip can increase the *WRKY38* mRNA abundance at 24 hours. Taken together, these data suggest that conversion of Pip to NHP may be required to alter *WRKY38* mRNA levels at later time points.

In Fig. S3, the white bar represents depleted biological processes. Can the authors clarify "depleted"?

Thank you for pointing this out, as we agree it is confusing. We have removed reference to "depleted". We have now described this as "underrepresented". We have also changed "enriched" to "overrepresented" to use consistent language. So all references to biological processes are referred to as "overrepresented versus underrepresented". This has been corrected in the text (page 9, lines 256 and 261; page 10, line 277; page 11, line 298; page 15, line 424) and figures (Figure 2, page 46 lines 1191 and 1194; Figure 3, page 47, lines 1204 and 1208) and Supplemental Figure S3.

In Fig. S7 legend, "D" should be "C".

We have now corrected this (new Figure S8, C).

We thank reviewer 1 for their thoughtful comments.

Reviewer #2 (Comments for the Author):

The study investigated the role of WRKY 70 in N-hydroxypipecolic acid (NHP) associated systemic acquired resistance (SAR) and its relationship with salicylic acid (SA) in SAR in Arabidopsis, especially at the early infection stage.

Strengths:
1. The study provided a detailed examination of the transcriptional responses to NHP over various time points, highlighting both early and late gene expression changes.
2. The use of the sid2-2 mutant (defective in SA biosynthesis) allows for the dissection of the relationship between NHP and SA, showing that NHP can induce gene expression changes independent of SA.
3. The study identified the key transcription factors (TFs) like TGA and WRKY that are involved in NHP signaling, enriching our understanding of the molecular mechanisms underlying SAR.
4. The research included functional validation by examining the role of specific WRKY TFs (e.g., WRKY70) in SAR, providing insights into the secondary transcriptional response driven by NHP.
5. The combination of gene expression analysis, TF binding site enrichment, and mutant phenotype characterization enhances the robustness of the findings.

Weaknesses:
1. While the study identified several WRKY TFs involved in NHP signaling, only WRKY70 is thoroughly investigated. Other TFs and their roles in NHP-induced SAR could be explored further or at least show some future insights.

As noted above, we agree that it would be interesting to study the function of other WRKY TFs in NHP signaling; however, this would be beyond the scope of this initial report. Our rationale is that the RNAseq study, screen, and characterization of one candidate resulting from the screen (WRKY70) provides a complete story and provides a rich data set for others to use to advance research in NHP signaling and SAR.

2. The findings sometimes reiterated known interactions between SA and JA signaling pathways, however, it needs to be further discussed how they function in SAR associated with WRKY70 and NHP.

We agree. We have provided discussion on these points in the discussion section (page 24, lines 712-724) and included a new figure (Figure 8; page 54, lines 1264-1284) to describe working models based on our findings and the literature.

3. Although the study covered multiple time points, the resolution between early and late responses could be improved to better understand the dynamics of NHP signaling.

We agree. The goal of our work was to provide an early snapshot (minutes to hours) of gene expression changes in response to NHP, which could be then used in follow-up experiments to better understand the dynamics of NHP signaling. We show in Figure 6B-D that *WRKY70* is required for the increase in mRNA abundance for a set of genes post-NHP treatment in adult plants at more fine-tuned timepoints, e.g, 6h, 9h, and 12h. Such studies will be necessary to apply the findings from the RNAseq seedling study to the behaviors in adult plants.

4. The study identified the correlations between TF binding sites and gene expression changes but did not delve deeply into the mechanistic details of how NHP influences TF activity and gene expression.

This is the focus of future work. Getting at mechanistic details will require the construction of new transgenic lines and mutants and tool development, which will take time and is beyond the scope of this initial study.

5. Method parts can be further improved by providing more details, such as the chemical compound preparation and related place for the purchase.

We have included the information for NHP chemical preparation, synthesis and company source. Please see page 26, lines 770-776.

6. It will be much helpful to provide a summary figure to show the related mechanisms and function of WRKY in NHP related SAR.

We have included a new figure (see Figure 8; page 54, lines 1264-1284), which describes our working models.

Overall, the study makes significant contributions to understanding the role of NHP in plant immunity, particularly in relation to SA and WRKY TFs at early stage, but could be strengthened b

This last sentence was cut off in the letter we received from the editor.

We thank reviewer 2 for their thoughtful comments.

31-Jul-2024
Prof. Mary Beth Mudgett
Stanford University
Department of Biological Sciences
Stanford, California 94305

MSID: PP2024-RA-00594R1
MS TITLE: Transcriptome analysis reveals role for WRKY70 in early N-hydroxy-pipecolic acid signaling

Dear Dr. Mary Beth Mudgett,

I am very pleased to inform you that your revised manuscript "Transcriptome analysis reveals role for WRKY70 in early N-hydroxy-pipecolic acid signaling" has been accepted for publication in Plant Physiology. Your manuscript will be checked for consistency with journal formatting and image standards by one of our Science Editors, who will contact you shortly. They may have a number of queries and suggestions for you. Please keep in mind that they work to increase the online visibility of your article and its attraction for readers from the widest of backgrounds. You will have the opportunity to review any revisions, and I hope you will work constructively with them to get the most out of your research. You will also have the opportunity to review all changes when you receive the author proofs for your article.

Once validated for publication, your article will be sent to a Science Editor for a final review of your manuscript and source files. After your final files are uploaded, your article will appear in the next available issue and the online Preview version of your article will be posted after your signed Copyright and Checklist forms and final source files are received.

Please be sure to include the following in your manuscript file:
Harvard style citations
List of all author contributions and funding information at the beginning of manuscript after the titles and author list.
List all supplemental materials associated with the article, with titles, at the end of the ms, before the references and acknowledgments section.
Complete Figure Legends for all figures appearing in the article listed before the Literature Cited.

Thank you and congratulations on your Plant Physiology paper!

Sincerely,

Editor Plant
Physiology

=====
FIRST AUTHOR PROFILES
=====

We recognize the hard work that goes into being First Author of a Plant Physiology paper by publishing a "First Author Feature" for each First Author alongside your paper.

If the first author(s) would like to submit information for a feature on Facebook and Twitter, fill out the form by clicking on the link below:

Plant Physiology First Author Feature Form: https://forms.office.com/r/ZBCGZfhm8F
If you cannot access the form through the link above, please contact Rachel at rbelsky@aspb.org.

To upload an image of yourself to accompany your profile, please put your photo in the correct journal folder at the link below. You must label your image: Author First Name Last Name Article Title.

First Author Profile Image Dropbox: https://www.dropbox.com/scl/fo/xz029p7qjr2jml2rp2kmq/h?dl=0&rlkey=6fkxcaqmxx35ap152b2osy1fz

=====
IMPORTANT REMINDER: PEER REVIEW REPORTS
=====

If you opted to publish a peer review report along with your article during the original submission process, it will be prepared by the editorial staff and publicly posted with your manuscript, inside the zip file that contains any other supplemental material. As a reminder, the peer review report is a public record of all comments from editors and reviewers, as well as your prior responses, as you received them in the decision letters for each draft of your manuscript. If you agreed to publish this report and have changed your mind, or are not sure if you selected this option, please contact the editorial office as soon as possible before signing the license agreement from our publisher.

-- Reviewer comments:
Reviewer #1 (Comments for the Author):

The authors have successfully addressed my concerns and I do not have further questions about the revised manuscript.

Reviewer #3 (Comments for the Author):

This manuscript reveals an new role of WRKY70 in early NHP signaling. The manuscript is clearly written and the main conclusions are supported by convincing evidence. I also believe that the authors have done a good job in adressing the comments from the editor and the two previous reviewers to a previous version of this manuscript. Accordingly, I have no further comments and recommend acceptance of this revised manuscript.
